# Response threshold variance as a basis of collective rationality

**DOI:** 10.1098/rsos.170097

**Published:** 2017-04-12

**Authors:** Tatsuhiro Yamamoto, Eisuke Hasegawa

**Affiliations:** Laboratory of Animal Ecology, Department of Ecology and Systematics, Graduate School of Agriculture, Hokkaido University, Sapporo 060-8589, Japan

**Keywords:** collective rationality, response threshold variance, collective decision-making

## Abstract

Determining the optimal choice among multiple options is necessary in various situations, and the collective rationality of groups has recently become a major topic of interest. Social insects are thought to make such optimal choices by collecting individuals' responses relating to an option's value (=a quality-graded response). However, this behaviour cannot explain the collective rationality of brains because neurons can make only ‘yes/no’ responses on the basis of the response threshold. Here, we elucidate the basic mechanism underlying the collective rationality of such simple units and show that an ant species uses this mechanism. A larger number of units respond ‘yes’ to the best option available to a collective decision-maker using only the yes/no mechanism; thus, the best option is always selected by majority decision. Colonies of the ant *Myrmica kotokui* preferred the better option in a binary choice experiment. The preference of a colony was demonstrated by the workers, which exhibited variable thresholds between two options' qualities. Our results demonstrate how a collective decision-maker comprising simple yes/no judgement units achieves collective rationality without using quality-graded responses. This mechanism has broad applicability to collective decision-making in brain neurons, swarm robotics and human societies.

## 1. Introduction

Determining the optimal choice among multiple options is necessary in various situations [1]. In collective decision-making, the final decision is made by collecting the many decisions of individual members [2–6]. How to achieve collective rationality is a major topic of interest in collective decision-making studies and has been approached from various perspectives [2–15]. In social insects, although an individual has a limited ability to assess the quality of options, the entire colony makes the optimal decision [9]. However, how a colony achieves such a collective rationality is not well understood [14,15].

A universal rule for making collective decisions is the majority decision. Many collective decision-makers, such as brains, social insects, group-forming bacteria, and human societies, adopt this rule to make a decision through quorum sensing [8,15]. There are several hypotheses to explain collective rationality in these systems, including positive feedback by stronger recruitment or more rapid recruitment to the best option (positive feedback/recruitment latency [14,15]) and best-of-*n* comparisons [14]. In social insects, more effective recruitment to the better option results in an increase in the number of visiting workers returning to the best option over time (i.e. positive feedback). These mechanisms inevitably require a difference in the response of individuals corresponding to the quality of an option (quality-graded responses) because a difference in the speed of recruitment results in a difference in the number of recruitments per unit time among options of different quality. The best-of-*n* requires the recruitment of nest-mates to the best option after a single individual has evaluated all options, and this behaviour represents a quality-graded response [14,15]. Thus, these mechanisms cannot explain collective rationality in a group in which any member can make only a simple yes/no response depending on the response threshold. Brains are thought to be such systems because the unit of a brain (a neuron) is thought to exhibit only a step-wise response to a response threshold [16]. A previous simulation of the collective decision-making of ants has demonstrated collective rationality in colonies with workers that exhibit only a step-wise response to a threshold stimulus but did not explain the reasons for this rationality [12]. Thus, the underlying mechanism of collective rationality by units that can make only a simple ‘yes/no’ response remains to be elucidated.

Approximately 90 years ago, two seminal studies showed that, in vision, a person can discriminate among differences in the strength of light by accumulating the ‘yes’ responses of visual cells with varied response thresholds [16,17]. We introduced this notion into collective decision research to show that variance in the response threshold can achieve collective rationality.

**Figure 1.**
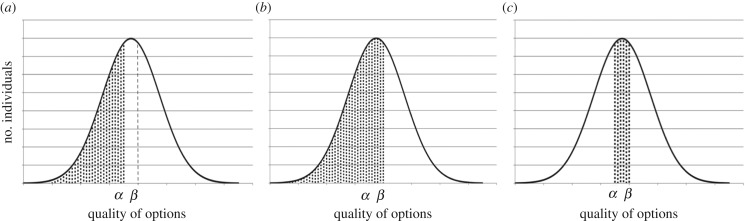
Majority-making mechanism for two options (each of a different quality) by a group comprising units with variable yes/no thresholds. Each option has a quality *α* or *β* (*α* < *β*), and the number of ‘yes’ response units for each option is the shaded area in (*a*) and (*b*), respectively. The number of ‘yes’ response units is larger for option *β* than for option *α* (the shaded area in (*c*)). As a result, option *β* (the better option) is always selected by a collective decision-maker using the majority decision.

The basic logic is as follows: in a collective decision-maker comprising many units, each of which can make only a step-wise ‘yes/no’ response depending on its response threshold (but not a quality-graded response), if the response threshold varies among the units of a group that evaluates the qualities of options, an option of higher quality will obtain a larger number of ‘yes’ responses (figure 1*a–c*). This conclusion holds for all possible pairs of options within the entire range of thresholds, irrespective of the distribution. If more than two options exist, a decision-maker can select the best option by repeating the same procedure. Therefore, a collective decision-maker can always choose the best option based on majority decision provided that the threshold distribution covers the range of the options' qualities. Thus, in principle, this mechanism guarantees the best choice among multiple candidates.

Variance among response thresholds would have an important role in achieving collective rationality through the mechanism described above. Figure 1 shows a schematic representation of this mechanism. A group of units that can make simple yes/no responses depending on their thresholds can quantify the qualities of the options available. There is a threshold variance among units (represented by normal distribution shown in figure 1*a–c*), and a unit provides a ‘yes’ response when the quality of an option exceeds its threshold. For two options (A and B), which have the qualities *α* and *β* (*α* < *β*), respectively, the number of ‘yes’ responses for option A is the shaded area shown in figure 1*a* and that for option B is the shaded area shown in figure 1*b*. There is an inevitable difference in the number of ‘yes’ responses between the two options (the shaded area in figure 1*c*) because the latter is always larger than the former. Therefore, a group can always choose the better option based on a majority decision.

Notably, in this decision-making process, units with lower thresholds than *α* do not contribute to forming the majority in the group because such units provide a ‘yes’ response to both options. Similarly, units with higher thresholds than *β* do not contribute to forming the majority because such units provide a ‘no’ response to both options. Thus, only units with thresholds between *α* and *β* influence the majority decision because, theoretically, these units respond ‘no’ to option A with value *α* but respond ‘yes’ to option B with value *β*. We verified this mechanism by determining the responses that occur in the above three threshold classes during a collective decision.

We will show that colonies of an ant species prefer the better option on the basis of this mechanism. We also discuss the relationships between our mechanism and previously proposed mechanisms based on observations.

## 2. Material and methods

### 2.1. Study organism and colony rearing

The study organism was *Myrmica kotokui*, a typical monomorphic ant. This species forms colonies in fallen rotten wood/trunks and moves deep into the ground during the winter. The average colony size is several hundred workers with a single queen. Details of the field collection and colony rearing have been described in a previous paper [18]. We collected six queenright colonies in June 2015 from the Tomakomai Experimental Forest of Hokkaido University in southwestern Hokkaido, Japan. All of the collected colonies contained a single queen and several hundred workers. From each colony, we randomly selected 56 workers to set up experimental colonies. All of the workers in the experimental colonies were individually marked at two points (on the thorax and abdomen) using Paint Markers™ (Mitsubishi, Inc., Tokyo, Japan). Combinations of eight colours for the thorax and seven colours for the abdomen enabled us to differentiate all the workers in each colony. Each of the six experimental colonies comprised 56 workers, a queen, eggs and larvae and was housed in an artificial nest (a plastic container with a plaster floor 3 cm deep; 30 × 22 × 6 cm). A single square chamber (10 × 7.4 × 1.5 cm) for the nest site was set at the centre of each container. The chamber was covered with a clear glass plate, and the ants were allowed to move freely in the container. The nest space was connected to an adjacent foraging area by a 1 cm tunnel. The colonies were reared under a temperature of 22°C and a natural light period. We fed the colonies with commercially available insect food (Nyusan Pro Jelly, Sapporo, Japan, Insect Pro Shop Hide) ad libitum.

### 2.2. Experiment 1

To test whether a worker exhibits a consistent threshold in response to the quality of a sucrose solution, we conducted an experiment after the colony had been starved for 3 days. A plastic case (4.9 × 7.4 cm) was set on the foraging arena of the colony, and 1 ml of sucrose solution (3.5% or 4.0%) was dropped into the case. Each worker was introduced into the case, and its behaviour was observed. When the worker began drinking the solution within 1 min of touching the solution with its antennae, we judged that the worker had responded to the concentration. If the threshold were to vary, three types of workers with different response patterns would be expected: (i) a worker responds to both concentrations, meaning that its threshold is lower than 3.5% (LOW), (ii) a worker responds to the 4.0% but not the 3.5% solution, meaning that its threshold is between the two concentrations (MID), and (iii) a worker does not respond to either of the concentrations, meaning that its threshold is higher than 4.0% (HIGH). We determined the responses of all of the workers to the two solutions in this experiment, and the experiment was repeated three times, with an interval of 3 days between experiments. The results for each colony are shown in table 1 and electronic supplementary material, tables S1–S3.

**Table 1.** Number of responding workers to those that arrived at each option in each class, and total number of responded workers to each option. In all six colonies, more workers were responded to the better option (4.0% sucrose solution) than to the worse option (3.5% sucrose solution).

	LOW		MID		HIGH		total no. responded workers	
colony	3.5%	4.0%	3.5%	4.0%	3.5%	4.0%	3.5%	4.0%
1	5/5	6/6	1/1	11/11	1/3	1/5	7	< 18
2	2/3	1/1	0/0	2/2	0/7	2/8	2	< 5
3	9/9	11/11	2/2	13/13	0/1	0/2	11	< 24
4	3/3	0/0	0/0	5/5	0/2	0/2	3	< 5
5	13/14	11/11	0/0	11/11	0/1	0/1	13	< 22
6	4/4	4/4	0/0	12/12	0/12	0/10	4	< 16

### 2.3. Experiment 2

We then conducted another experiment using the monitored workers. A colony was provided with two different concentrations of sucrose solution (3.5% or 4.0%). In two plastic cases (each 3.7 × 6.7 cm), solutions containing 3 ml of either 3.5% or 4.0% sucrose were placed in the foraging arena of each colony at an equal distance (8 cm) from the nest entrance. Each of the two options was placed at a location different from the usual food site to minimize the effects of pre-deposited pheromone trails to the usual food site. Food at the usual food site was removed before the start of the experiments. We recorded the ants’ behaviour as they arrived at each option using a video recorder (HC-V720M, Panasonic, Tokyo, Japan); when a worker arrived at an option, we recorded its response. When it started to drink the sucrose solution within a minute of touching the solution with its antennae, we judged that the worker had responded. The number of responding workers was counted 15 min after the start of the experiment to eliminate the effects of subsequent recruitment. The option that received the greatest number of responses from the ants was considered the preferred option. On the basis of the data, (i) the number of arriving workers, (ii) the number of responding workers, and (iii) the proportion of responding workers within the arriving workers were compared between the two options for each threshold class (LOW, MID or HIGH).

### 2.4. Statistical analysis

The ratio of the number of colonies that preferred the better option to those that preferred the worse option was tested for the difference from 1 : 1 using a binomial test. The numbers of arriving workers for each option were compared using Wilcoxon's signed rank sum test for the combined data over all colonies. The differences in the numbers of workers arriving at the different options and the differences in the proportion of workers arriving at each option were also examined using Wilcoxon's signed rank sum test. All statistical analyses were conducted using R (v. 3.2.3).

## 3. Results

In experiment 1, of the 336 examined workers, 307 showed a consistent response during the experimental period. The 29 workers that did not show a consistent response were removed from further experiments. Among the 307 consistent workers, the numbers of individuals for each threshold class were as follows: LOW = 104, MID = 119 and HIGH = 84. These results demonstrate that an individual worker usually has a consistent threshold and that the thresholds vary among workers.

**Figure 2.**
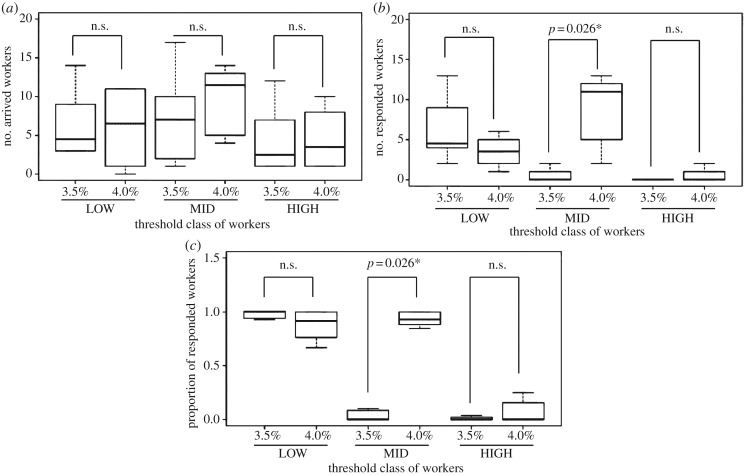
Responses of workers belonging to three threshold classes (LOW, MID, HIGH) to the quality of two options (3.5% and 4.0% sucrose solutions). The black horizontal line indicates the median. Boxes delimit the first and third quartiles, and whiskers show the range. An asterisk indicates a significance level of *p* < 0.05. (*a*) Significant differences were not observed in the number of workers arriving at each option in each class. (*b*) Significant differences were observed only in the numbers of workers responding to the sucrose concentrations in the MID class. (*c*) The proportion of responding workers among the arriving workers was significantly different only in the MID class.

We then conducted a binary choice experiment between two options with different quality values (3.5% and 4.0% sucrose solutions). All six colonies preferred the better option (table 1), and the bias was significantly different from 0.5 (for the combined data; binomial test, *n* = 6, *p* = 0.031). Because all six colonies showed similar response patterns (table 1; electronic supplementary material, tables S1–S3), the data were combined for the following statistical analyses. The number of workers that arrived was 108 for the 3.5% option and 122 for the 4.5% option, but this difference was not significant (Wilcoxon signed-rank test, *V* = 2, *n* = 6, *p* = 0.237). Among the three threshold classes, significant differences were not observed in the number of workers arriving at each of the options (figure 2*a*). These results indicated that the collective preference of the colonies was not generated by asymmetric allocations of the workers to each option. In addition, we were able to reject the possibility of an effect of pheromone trails deposited before the experiment because if there were a stronger pheromone trail to the better option than to the worse option, more workers would have visited the former, but the observations did not support this hypothesis (Wilcoxon signed-rank test, *V* = 2, *n* = 6, *p* = 0.237). Thus, we rejected the effects of pre-deposited pheromone trails on the colonies' preferences (see the Material and methods section for a discussion of how the effects of pre-deposited pheromone trails were avoided).

Figure 2*b* shows the number of workers that responded to each of the two options (3.5% and 4.0% sucrose solutions). According to the predictions of our hypothesis, a significantly larger number of workers responded to the better option only in the MID class (Wilcoxon signed rank sum test, *V* = 0, *n* = 6, *p* = 0.026). Figure 2*c* shows the proportions of workers that responded to each option among those that arrived at each option. The results showed that a significantly larger proportion of the workers in the MID class responded ‘yes’ to the better option but ‘no’ to the worse option (Wilcoxon signed rank sum test, *V* = 0, *n* = 6, *p* = 0.026). Most workers in the HIGH class responded ‘no’ to both options, and most in the LOW class responded ‘yes’ to both options (figure 2*b,c*). These results clearly show that the predictions of our hypothesis are supported. The individual responses of the workers in the MID class had a substantial influence on the majority preference of the entire colony (table 1 and figure 2*b*,*c*).

It is important to note that because, in theory, our mechanism works regardless of the options’ qualities and the threshold distribution, a single experiment was sufficient to prove the hypothesis. If we set another pair of options, each with a different quality, ant colonies are predicted to always prefer the better option provided that their threshold distribution covers the range of the options’ qualities.

## 4. Discussion

Our results showed that individual *Myrmica kotokui* workers exhibit consistent thresholds that vary among workers. In addition, our study clearly demonstrated the possibility of a collective decision-maker making an optimal choice on the basis of the threshold variance among multiple options. Several studies have shown threshold variances among workers in social insects [18–20] (this study), and this phenomenon is thought to realize optimal task allocations in groups that do not have dominant members [21] because it enables a colony to allocate a required number of workers for each task on demand. Recently, this system has been shown to guarantee the long-term sustainability of a colony, although it also results in a loss of short-term productivity [22]. However, the role of this threshold variance in collective decision-making has been overlooked. A previous simulation study in which a threshold variance among workers was introduced into the model to simulate a real social-insect colony has indicated that the optimal choice is made [12], although the underlying mechanisms remained unexplained. In addition, a recent review of the inter-individual variability of the workers of a colony of social insects does not mention the role of threshold variance in the collective decision-making process [23]. Thus, to our knowledge, the results presented here are the first to demonstrate the key role of threshold variance in collective rationality.

Previously, collective rationality in social insects was thought to occur because of quality-graded behaviour among the workers [23]. All previous hypotheses (positive feedback, including recruitment latency and the best-of-*n*) require workers to be able to recruit with different strengths to options with different quality values. It is well known that honeybee scouts perform stronger and longer waggle dances when they visit a good option than a poorer option [24]. This quality-graded response results in the recruitment of more workers to better options, and the best choice is made by the majority decision with a quorum [15]. The same procedure has also been reported in ants [8,14]. However, even in these cases, the outcome of whether a scout demonstrates recruitment behaviour (a waggle dance in honeybee and tandem running in ants) may be controlled by a response threshold depending on the quality of the option. In fact, it has been reported that a considerable proportion of honeybee scouts do not dance after visiting an option (see fig. 3 in [25]), thus suggesting that, in such bees, the quality of the visited option does not reach the threshold, and there is likely to be a response-threshold variance among the scouts in response to the options' qualities because other scouts perform dances in response to the same option (see fig. 3 in [25]).

The occurrence of our mechanism in the honeybee is also suggested by observations reported in several studies. Dancing was performed by 80.5% (33 of 41) of scouts after visiting a high-quality option but was performed by a significantly smaller proportion of scouts (48.6%, 18 of 37) after visiting a low-quality option (*χ*^2^-test, *χ*^2^ = 8.51, *p* < 0.004 [24]). A larger number of scouts (33 > 18) perform dances in response to the better option on the basis of the response-threshold variance among the scouts, and the number of workers recruited by these dances also thereby depends on the thresholds. Additionally, in nest hunting in *Temnothorax* ants, the proportions of scouts that show recruitment behaviour differ according to the qualities of the options [26,27]. The acceptance rate of a candidate nest has been found to be higher for a high-quality option than for a low-quality option in two different studies (0.053 > 0.034 for the thickness of a candidate nest and 0.032 > 0.013 for the darkness of an option (see table 1 in [24])). The acceptance rate is higher for the high-quality option than for the low-quality option regardless of the urgency of the emigration (see the middle panel in fig. 4 in [26]). Therefore, there is a possibility that the ‘yes/no’ mechanism underlies collective decision-making even in social insects. Of course, quality-graded responses may also explain the above differences, and thus the means by which one (or both) of the mechanism(s) realizes these differences should be tested in future studies. Notably, the two mechanisms may work simultaneously to cause the observed differences in the numbers of workers that respond to options of different qualities.

However, the current mechanism is not mutually exclusive with the previously proposed mechanisms. The possibility exists that the difference in the number of responding workers caused by our mechanism may be enhanced or corrected by the previously proposed mechanisms in nature. In fact, the above-mentioned honeybee study [24] has also shown that scouts that respond to the high-quality option perform stronger dances than those that visit the low-quality option. The previously proposed mechanisms appear to enhance the correctness of the majority decision after the initial judgement on the basis of our mechanism. In some cases, these complementary mechanisms may correct an initial error that may occur under our mechanism, especially when the number of scouts is small. For example, if eight or 10 scouts visit high- and low-quality options, respectively, and if seven of eight (87.5%) respond ‘yes’ to the former option, this number would be smaller than eight of 10 ‘yes’ responses (80%) to the latter option. In such a case, although a larger proportion of scouts responded ‘yes’ to the former option under the current mechanism, a wrong decision would be made under the immediate decision because the number of scouts is too small to make a robust correct decision. If the number of scouts is sufficiently large, the proposed mechanism always achieves the best choice; however, in nature, only a limited number of scouts can be sent to each option. In cases in which the initial judgement based on the yes/no mechanism is wrong, quality-graded recruitment would correct the initial errors if enough time were devoted to the decision-making [28].

The yes/no mechanism is not mutually exclusive with the quality-graded mechanisms. Rather, the yes/no mechanism underlies the collective rationality as a basis, but both mechanisms together guarantee corrective rationality. Because collective rationality is necessary for the survival of group-living organisms, it is worth examining further how the two mechanisms work together in rational collective decision-making. We are currently examining collective rationality in a situation in which the yes/no mechanism cannot make a rational selection using only the LOW class in the selection. Such studies will elucidate the roles of the yes/no mechanism and the quality-graded mechanism in collective rationality in social insects.

This study assumed a majority preference 15 min after the start of the experiment, which was too short a time period to perform a significant amount of recruitment. In fact, we did not identify any arrived worker that showed recruitment behaviour (tandem running or depositing trail pheromone). Thus, the observed results could not be explained by the previously provided mechanisms. As mentioned above, the effect of pre-deposited pheromone trails can also be rejected. However, all six colonies examined succeeded in preferring the better option in the binary choice experiment. Thus, our mechanism appears to be used in the initial stage of the collective decision-making and may enable a colony to achieve a rapid collective rationality.

Notably, our mechanism can explain the collective rationality of brains that are thought to comprise neurons that can only make simple ‘on/off’ responses on the basis of their response thresholds [29]. If this is true, any hypothesis that requires quality-graded responses cannot explain the brain's collective rationality. Previous studies on brain decisions in monkeys have shown that in the brain region where the quality of an option is assessed, more neurons fire as the quality of an option increases, and the rate of firing in response to an option's quality is not influenced by the existence of other options [30–32]. These findings can be explained using the ‘yes/no’ responses of only a single neuron. Thus, to propose another mechanism for the brain's collective rationality based on quality-graded responses, it must be shown that a single neuron is capable of making a quality-graded response to changes in a stimulus. Possibilities include the existence of non-spiking neurons [33] and the high-frequency repetitive firing of a neuron [34]. This is an issue that should be elucidated in further studies.

At present, almost no information is available on the nature of the ‘yes/no’ mechanism. In addition, the factors that affect the performance of a yes/no decision-maker are not well understood. The mechanism described here can be applied to various fields, such as brain science, behavioural science, swarm robotics and consensus decision-making in human societies. Therefore, it will be fruitful to elucidate the nature of collective decision-making processes with regard to this mechanism in future studies.

## Supplementary Material

Number of workers in each threshold class and raw data of the experiment 2

## Data accessibility

All the data used are provided in table 1 in the text and electronic supplementary material, tables S1–S3.
