# Supplementary material for: Response threshold variance as a basis of collective rationality
Source: R Soc Open Sci. 2017 Apr 12;4(4):170097. doi: 10.1098/rsos.170097 (PMC5414273; doi:10.1098/rsos.170097)
Supplement: Number of workers in each threshold class and raw data of the experiment 2 [file rsos170097supp1.docx]

**Supplementary Materials**

Table S1. Number of individuals classified into the three threshold classes. LOW includes the individual ants that responded to both option qualities (3.5% or 4.0% sucrose solution). MID includes the individual ants that responded to 3.5% but not to 4.0%. HIGH includes the individual ants that did not respond to either of the options. VARIABLE indicates the ants did not show a consistent response during the 3 repeated experiments. ERROR indicates the individuals that responded to 4.0% but not to 3.5%.

| Class | LOW | MID | HIGH | VARIABLE | ERROR | Total |
| --- | --- | --- | --- | --- | --- | --- |
| Threshold | T<3.5% | 3.5%<T<4.5% | 4.5%<T | not consistent  throughout 3 tests | Responded to 3.5% but not to 4.0% |  |
| Colony 1 | 13 | 26 | 13 | 3 | 1 | 56 |
| Colony 2 | 5 | 13 | 30 | 5 | 3 | 56 |
| Colony 3 | 25 | 23 | 4 | 4 | 0 | 56 |
| Colony 4 | 9 | 15 | 28 | 4 | 0 | 56 |
| Colony 5 | 25 | 23 | 4 | 4 | 0 | 56 |
| Colony 6 | 27 | 19 | 5 | 5 | 0 | 56 |
| Total | 104 | 119 | 84 | 25 | 4 | 336 |

Table S2. Number of workers classified into the three threshold classes in each of three repeated measurements. Abbreviations are the same as those in Table S1.

|  | Measurement | | | | | | | | | | | |
| --- | --- | --- | --- | --- | --- | --- | --- | --- | --- | --- | --- | --- |
|  | 1st check | | | | 2nd check | | | | 3rd check | | | |
| Colony | LOW | MID | HIGH | ERROR | LOW | MID | HIGH | ERROR | LOW | MID | HIGH | ERROR |
| 1 | 14 | 27 | 13 | 1 | 13 | 28 | 14 | 0 | 14 | 28 | 13 | 0 |
| 2 | 5 | 18 | 32 | 1 | 5 | 15 | 34 | 2 | 5 | 17 | 34 | 0 |
| 3 | 28 | 24 | 4 | 0 | 28 | 24 | 4 | 0 | 25 | 27 | 4 | 0 |
| 4 | 10 | 15 | 31 | 0 | 9 | 17 | 30 | 0 | 10 | 18 | 28 | 0 |
| 5 | 28 | 24 | 4 | 0 | 28 | 24 | 4 | 0 | 25 | 27 | 4 | 0 |
| 6 | 30 | 20 | 6 | 0 | 30 | 21 | 5 | 0 | 29 | 21 | 6 | 0 |

Table S3. Number of workers that arrived to each option after 15min from the start of experiment. The numbers are shown for each threshold class. Abbreviations are the same as those in Table S1.

|  | LOW | | MID | | HIGH | | Total | |
| --- | --- | --- | --- | --- | --- | --- | --- | --- |
| Colony | 3.5% | 4.0% | 3.5% | 4.0% | 3.5% | 4.0% | 3.5% | 4.0% |
| 1 | 5 | 7 | 10 | 13 | 3 | 5 | 18 | 25 |
| 2 | 3 | 1 | 2 | 4 | 7 | 8 | 12 | 13 |
| 3 | 9 | 11 | 6 | 14 | 1 | 2 | 16 | 27 |
| 4 | 3 | 0 | 1 | 6 | 2 | 2 | 6 | 6 |
| 5 | 14 | 11 | 8 | 11 | 1 | 1 | 23 | 23 |
| 6 | 4 | 6 | 17 | 12 | 12 | 10 | 33 | 28 |
| Total | 38 | 36 | 44 | 60 | 26 | 28 | 108 | 124 |

Table S4. Number of responded workers to the workers that arrived to each option. Total is the total number of responded workers to each option. In LOW class, the most workers responded to both the options. Contrary, almost no workers responded to both the options in HIGH class. The responses by workers in MID class substantially influenced the preference of the colonies. In all the 6 colonies, the better option (4.0% sucrose solution) was preferred by more workers. Abbreviations are the same as those in Table S1.

|  | LOW | | MID | | HIGH | | Total | | |
| --- | --- | --- | --- | --- | --- | --- | --- | --- | --- |
| Colony | 3.5% | 4.0% | 3.5% | 4.0% | 3.5% | 4.0% | 3.5% |  | 4.0% |
| 1 | 5/5 | 6/6 | 1/1 | 11/11 | 1/3 | 1/5 | 7 | < | 18 |
| 2 | 2/3 | 1/1 | 0/0 | 2/2 | 0/7 | 2/8 | 2 | < | 5 |
| 3 | 9/9 | 11/11 | 2/2 | 13/13 | 0/1 | 0/2 | 11 | < | 24 |
| 4 | 3/3 | 0/0 | 0/0 | 5/5 | 0/2 | 0/2 | 3 | < | 5 |
| 5 | 13/14 | 11/11 | 0/0 | 11/11 | 0/1 | 0/1 | 13 | < | 22 |
| 6 | 4/4 | 4/4 | 0/0 | 12/12 | 0/12 | 0/10 | 4 | < | 16 |
